# The Luganda Edinburgh Postnatal depression scale: cross-cultural adaptation and validation for prenatal screening of depression in a Ugandan sample

**DOI:** 10.4314/ahs.v24i4.28

**Published:** 2024-12

**Authors:** David Christopher Mukasa, Sam Ononge, Imelda Namagembe, Josaphat Byamugisha, Musa Sekikubo, Mark Muyingo, Noeline Nakasujja

**Affiliations:** 1 Department of Obstetrics and Gynaecology, Makerere University, Kampala, Uganda; 2 Department of Psychiatry, Makerere University, Kampala, Uganda; 3 Mulago Specialized Women's and Neonatal Hospital, Kampala, Uganda; 4 University hospital, Makerere University, Kampala, Uganda; 5 Neogenesis Fertility Centre, Kampala, Uganda

**Keywords:** Mental health, prenatal depression, Edinburgh Postnatal Depression Scale, Luganda

## Abstract

**Background:**

Depression affects approximately 364 million people globally. Prenatal depression affects between 26.3% and 32.9% of mothers in Africa. Opportunities for prenatal screening are missed. The gold standard diagnostic, the Diagnostic and Statistical Manual of Mental Disorders-Fifth edition (DSM-5) criteria for Major Depressive Disorder (MDD) has higher technical requirement. There is inadequate information on locally adapted and validated user-friendly screening tools in Uganda.

**Objective:**

To Adapt and validate the Luganda Edinburgh Postnatal Depression Scale (EPDS-L) for screening prenatal depression at Kawempe National Referral hospital (KNRH).

**Methods:**

Cross-sectional study in KNRH using International Society for Pharmacoeconomics and Outcomes Research guidelines for adaptation and quantitative approaches for the validation. Consecutive sampling until the desired sample of 100, all participants responded to both EPDS-L and DSM-5 criteria for MDD. Reliability demonstrated using Cronbach's alpha coefficient, while validity was demonstrated by sensitivity, specificity, Negative Predictive Value (NPV), Positive Predictive Value (PPV) and Area-Under-the-curve (AUC).

**Results:**

EPDS-L had Cronbach's-Alpha of 0.8515. At cut-off of 13, sensitivity was 62.86%, specificity-100%, PPV-100% and NPV-83.3%. AUC was 0.99. Performance was better at cut-off of 10, with sensitivity-97.14% and specificity-98.46%.

**Conclusion:**

The EPDS-L is reliable at cut-off of 13 but performs even better at cut-off of 10.

## Introduction

According to the WHO, mental health is a state of well-being in which an individual realizes his or her own abilities, can cope with the normal stresses of life, can work productively and is able to make a contribution to his or her community 1. Depression is one of the mental disorders characterized by presence of at least 5 symptoms based on DSM-5 criteria for Major depressive disorder in presence of any of the following: persistent sadness and a lack of interest or pleasure in previously rewarding or enjoyable activities 2. Studies have identified several risk factors for prenatal depression. These have been grouped into social factors, obstetric factors and psychiatric factors^3^. These include extremes of age, low education level, low family support, unintended pregnancy, complications in prior pregnancy among others 4. Despite sufficient evidence of the significant burden of prenatal depression (3), the continuum of care remains insufficient. There is inadequate screening for the condition. This may be attributed to the high technical requirements to diagnose this condition among prenatal mothers 5.

The known diagnostic gold standard (the DSM-5 criteria for Major Depressive Disorder) requires further psychiatric training for one to comfortably use and interpret findings 6, 7. There are several non-native screening tools for screening depression, however none is locally adapted and validated for use in prenatal depression in Uganda. Local adaptation and validation enables application of the tool regardless of level of psychiatric training. This enhances case identification and linkage to care.^8^. Untreated prenatal depression has been associated with adverse pregnancy outcomes 9 as well as negative long term sequalae in the offspring 10. Although prenatal depression poses immense negative impact on pregnancy, it remains rather neglected among women in Low to Middle Income Countries (LMIC). In Uganda, a policy brief on screening of depression in maternal care was presented in 2017 11, however not much has been implemented in Kawempe National Referral Hospital. Perhaps this is due a knowledge gap among health care providers in identifying the symptoms and screening mothers.

The Edinburgh Postnatal Depression scale (EPDS) originally developed in the UK and validated among 84 mothers 12 has been used in several settings to screen for prenatal depression and has demonstrated sufficient psychometric properties 13, 14. The tool was originally used to screen postpartum depression 12, but subsequently became applied among prenatal mothers as well. It is a 10-question tool, with a score of 0-3 for each question. Adaptation and validation of the Luganda version of the EPDS will enhance screening through roll out of an easily applied, timesaving, user-friendly tool that can be applied by even the lower level health care provider. Inclusion of prenatal depression screening in maternal care falls in line with Sustainable Development Goal 3 (target 7) 15; Objective 4 (section 4) of National Development Plan 3 (NDP3) 16 and Objective 3.10 of the Ministry of Health strategic plan 2020-2025 17. The purpose of this study was to adapt and validate the Luganda version of the Edinburgh Postnatal Depression scale for screening prenatal depression among mothers attending the antenatal clinic at KNRH.

## Materials and methods

### Study design

This was a cross-sectional facility-based study in Kawempe National Referral hospital.

### Research approaches

This study was conducted using the International Society for Pharmacoeconomics and Outcomes Research (ISPOR) taskforce guidelines for adapting the EPDS-L, and quantitative approaches for validating it.

### Study setting

The study was facility-based and conducted at Kawempe National Referral Hospital antenatal clinic. Kawempe National referral hospital is situated approximately 12 Kilometers from the Kampala city Centre 18. The hospital is found in the central part of Uganda, Buganda, where Luganda is the main dialect and majority understand the language. The hospital provides tertiary level maternity, obstetric and gynecological services to the urban poor and average income earners who walk in. The hospital also receives referrals from peripheral health units in Greater Kampala and countrywide. KNRH also serves as a Teaching hospital for Makerere University. The antenatal clinic of KNRH has an attendance of approximately 200 patients per day, and the clinic happens thrice a week^18^, which translates into an attendance of 2400 mothers per month.

### Phase 1: Adaptation of the EPDS-L for screening of prenatal depression in the antenatal clinic of KNRH

The English version of the EPDS was translated into Luganda (EPDS-L). Standard procedure for translation of tools, as described by the International Society for Pharmacoeconomics and Outcomes Research (ISPOR) taskforce was followed 19. This process involved 9 steps as described below:

#### i. Preparation

The EPDS is a known tool developed for screening perinatal depression. The tool has also been validated of screening prenatal depression in several settings and has demonstrated sufficient psychometric properties. The tool is available for use as long as copyright is acknowledged.

#### ii. Forward translation

Two Luganda language experts from the School of Languages, Literature and Communication, College of Humanities and Social sciences at Makerere University were requested to independently forward translate the English version of the EPDS into Luganda. During this process, the forward translators were asked to provide details and clear intention of each question in the EPDS. The two translations were intended to enhance detection of divergence in interpretation, errors and reduce bias. The Luganda language was chosen on the basis of the location of KNRH within Buganda, and majority of the population that seek services from the hospital understand Luganda.

#### iii. Reconciliation

A translation panel was constituted. This panel included the Principal Investigator, the Co-Investigators, the two forward translators and an independent native Luganda speaker not initially involved in the study. The panel reconciled the 2 versions of forward translations made and reached consensus.

#### iv. Back translation

The reconciled Luganda version of the EPDS (EPDS-L) was then be back-translated into English. This was undertaken by another group of two Luganda /English language experts from the School of Languages, Literature and Communication, College of Humanities and Social sciences at Makerere University. These back translators were not provided with any information about the initial English version of the EPDS and were not involved in the initial forward translation.

The back translation was done to ensure quality of the initial forward translation. This was done to ensure that there was no divergence between the back translated version and the original English EPDS version.

#### v. Harmonization

A meeting involving the Principal Investigator and back translators was held to reach consensus on the available back translations done.

#### vi. Cognitive debriefing

Five Luganda-speaking mothers available at the antenatal clinic on clinic day were consecutively selected and requested to respond to the harmonized EPDS-L to assess the comprehensibility of the tool. These five included mothers who speak Luganda as a first language, as well as those who had another language as their first, but understood Luganda. A Comprehension Assessment checklist was filled to completion for each of the 5 mothers based on their ability to read and explain the meaning of each of the 10 items in the harmonized Luganda version.

#### vii. Review of cognitive debriefing results and finalization

The Principal Investigator reviewed the findings of the cognitive debriefing and further fine-tuned the EPDS-L together with the Co-investigators.

#### viii. Proof reading

All errors in writing the EPDS-L were identified and corrected.

#### ix. Final report

The final EPDS-L developed was presented to the entire research team.

### Phase 2: Validation of the EPDS-L in screening prenatal depression in the antenatal clinic of KNRH

#### Population

##### Study population

The study population included prenatal mothers.

Target population

Prenatal mothers attending antenatal care in Uganda.

##### Accessible population

Prenatal mothers that were present in the antenatal clinic of Kawempe National Referral hospital who met the eligibility criteria.

### Eligibility criteria

#### Inclusion criteria

Mothers who could express themselves in Luganda were included. Mothers with known history of depression were also included, as long as they had insight and were stable enough to participate. These were identified as clients who had a history of sadness that had required medical treatment. Mothers less than 18 years of age were also included as emancipated minors.

#### Exclusion criteria

Prenatal mothers found to be have participated in similar studies involving psychiatric assessment were excluded as this would biase their responses. However, none of the eligible mothers had done, hence none were excluded based on that history.

### Sample size

The sample size for Patient Reported Outcomes (PRO) may be determined using the item to participant ratio 20. Given that the EPDS is a 10-item tool, an item to participant ratio of 1:10 was used 20, 21. This translated into a sample size of 100 participants. Similar to the validation of the Bangla version of the Edinburgh Postnatal Depression scale, a convenience sample of 100 was used^22^.

### Data collection tools

The EPDS has been validated in different settings 23-25. This scale has also been used to screen for postpartum depression in Uganda^26^, ^27^. The Cronbach's Alpha coefficient of the EPDS for screening prenatal depression in Rwanda was 0.89 28. The EPDS comprises 10 items that describes different depressive symptoms. Each item is awarded a score on a scale of 0 to 3.0 implies the symptom is absent, while 3 implies severity of the symptom. The maximum score awarded is 30. Participants who scored 13 and above were classified as depressed in a prior study done in Uganda 4. The adapted EPDS-L was administered to eligible participants. Following completion, the DSM-5 criteria, which is the standard diagnostic criteria for major depressive disorder was administered by the same research assistants who were qualified psychiatric nurses trained on the protocol. The DSM-5 criteria for MDD was used as the gold standard for diagnosis. The criteria comprise 9 major items that signify the depressive symptoms. Presence of five of the symptoms within a fortnight is diagnostic. At least one of the 2 core depressive symptoms must be present among the five. These are persistent sadness and a lack of interest or pleasure in previously rewarding or enjoyable activities.

### Data collection

Demographic data was collected by 2 trained research assistants who were also qualified psychiatric nurses and had been trained on the protocol. Upon completion, EPDS-L was then administered. The study team ensured that the data collection tools were filled to completion. The EPDS-L comprised 10 questions, each with a score of 0 to 3, with the maximum total score of 30. Any score beyond 13 was considered as depression. Following completion of the EPDS-L, the research assistants administered the DSM-5 Diagnostic Criteria for Major depressive disorder to all participants to elicit depressive symptoms. The DSM 5 criteria for diagnosis of Major depressive disorder was used as the gold standard. Presence of five or more symptoms, with criterion 1 or 2 or both among the five was considered DSM-5 positive for depression.

### Procedure

Following ethical approval and administrative clearance, the Principal Investigator introduced the study to the Sister in-charge of the antenatal clinic at KNRH. Routinely, mothers were registered on arrival to the antenatal clinic and were attended to in order of registration. The study team accessed the antenatal register and selected potential participants based on already known risk factors of prenatal depression. Based on prior research by Ndege et al in Mulago, factors found to be significantly associated with prenatal depression included extremes of age less than 20 or advanced maternal age, low education level of primary or less, unintended pregnancy, complications in prior pregnancy low, family support among others 4. All these details had been captured into the antenatal register on arrival. The study team easily accessed this register throughout the period of data collection. This register was used to risk stratify potential participants before approaching them. Both low-risk and high-risk mothers were approached for inclusion. A total of 121 mothers were approached for inclusion. Twenty-one were however not recruited due to decline to consent to participate, too sick to participate or failure to locate them within the antenatal clinic. Consecutive recruitment into the study following written informed consent was done. Mothers were recruited until the convenience sample size of 100 participants was achieved. Written informed consent was obtained by the principal investigator or research assistants following comprehensive explanation on the purpose of the study, benefits, and risks and thereafter requested for their participation. Eligible clients who agreed to participate signed the consent form by writing their name and signature and were subsequently assigned a study number. Participant consent was witnessed by the research assistants who appended their names and signatures on the consent forms. Participants were interviewed on each of the three antenatal clinic days per week. These were Tuesday, Wednesday and Thursday from December of 2022 to January 2023. Approximately 7 to 9 mothers were recruited per day from 8am to 4pm. Data collection was done in designated isolated places in the antenatal clinic for participant privacy. All study participants responded to the demographic data questionnaire, the EPDS-L and lastly DSM-5 criteria for MDD administered. Data tools were checked thoroughly to ensure completeness before participants left the data collection stations. All study participants were then supported to receive their routine antenatal care.

### Data management

Face and content validity of the EPDS-L was established during the pre-testing phase on mothers in the same antenatal clinic. Quantitative data was checked for completeness before the participant left, coded and entered into EPIDATA software. Double entry of data was done, completeness was ensured. The data was then exported to STATA version 15 for analysis.

### Data analysis

Descriptive statistics for respondents mainly included proportions and percentages and were summarized in Table 1 and in text. The reliability of the EPDS-L was determined using the Cronbach's alpha coefficient. The concurrent criterion validity was demonstrated through cross tabulating the EPDS-L score against the actual prenatal depressive status based on the DSM-5 criteria as the gold standard. From this, the maximum sensitivity, specificity, Positive Predictive Value (PPV), and Negative Predictive Value (NPV) of the EPDS-L were calculated. Additionally, to establish the cut off points above which the EPDS-L can be reliably used to screen for prenatal depression, the levels of sensitivity and 1-specificity of the EPDS-L were assessed. Furthermore, the Area Under the Receiver Operating Characteristic curve (AUROC) was plotted to confirm whether the cut off level of 13 is optimum to define threshold level for depressive symptom using the EPDS-L.

**Table 1:** Descriptive Statistics of prenatal mothers attending the antenatal clinic at Kawempe National referral hospital

*Variable*	Category	Percentage (%)
*Age in years*	14-19	3
	20-29	51
	30-39	29
	40	17
*Marital Status*		
	Married	83
	Not Married	17
*Highest level of education*		
	Never educated	1
	Primary level	20
	Secondary Level	55
	Tertiary Level	24
*Household Income Level(monthly)*		
	Less than UgX 100,000	67
	UgX.100,000 - 500,000	13
	More than UgX 500,000	20
*Felt supported (physical/financial/emotional)*		
	Yes	80
	No	20
*Gravida*		
	Prime	19
	2-4	55
	5 or more	26
*Intention of current pregnancy*		
	Wanted	74
	Unwanted	26
*Weeks of amenorrhea*		
	First Trimester	3
	Second Trimester	39
	Third Trimester	58

### Ethical considerations

Approval to conduct the study was obtained from School of Medicine Research and Ethics committee of Makerere University (Ref Number-Mak-SOMREC-2022-459). Written informed 394 consent was obtained from all study participants prior to being enrolled in the study. Participation was entirely voluntary.

## Results

In this study, a total sample size of 100 mothers was considered and had their questionnaires completed, thereby achieving 100% response rate. Half the mothers (51%) were between the age group 20 to 29 years, a third (29%) between 30-39 years, about a fifth (17%) were ≥40 years, and only a few (3%) were between the age group of 10-19 years as shown in Table 1.

Majority (83%) were married, and just over half (55%) had achieved Secondary level education.

Socioeconomically, two-thirds (67%), had a monthly household income of less than 100,000 Ugandan shillings per month, while a fifth (20%) had more than 500,000 Ugandan shillings (Table 1).

Based on the DSM-5 criteria for Major depressive disorder, about two-thirds screened (65%) did not have depression while the other third (35%) had it. This is illustrated in Table 2 and Table 3.

**Table 2:** Sensitivity and specificity analysis of the adapted Luganda version of the EPDS

*EPDS-L*	*Gold standard (DSM 5)*		*Total*
	Has Depression	No Depression	
***Has Depression***	22	0	22
***No Depression***	13	65	78
*Total*	35	65	100

**Table 3:** Psychometric property analysis of the adapted Luganda version of the Edinburgh Postnatal Depression scale

Variable	Probability	Percentage	[95% Conf. Inter.]
Sensitivity	Pr(+∣ D)	62.86%	[53.39% - 72.33%]
Specificity	Pr(-∣∼D)	100.00%	[100.00%-100.00%]
Positive predictive value	Pr(D∣+)	100.00%	[100.00%-100.00%]
Negative predictive value	Pr (∼D∣-)	83.33%	[76.03% - 90.64%]
Prevalence	Pr(D)	35.00%	[25.65% - 44.35%]
**D defined as True Depression**		**∼D defined as true non-depression**	

One hundred (100) mothers were screened for depression using the EPDS-L. About two-fifths (48%) had a score of 5-12, about a third (30%) with a score of 1-4, while a fifth (22%) screened positive with a score of 13 or more.

### Reliability of EPDS-L

The reliability of the EPDS-L was determined using the Cronbach's alpha coefficient. Ten (10) items were assessed for internal consistency and the Cronbach's alpha coefficient was 0.8515 with inter-item covariance of 0.394. Cronbach alpha values of 0.7 or higher indicate acceptable internal consistency and therefore, the EPDS-L was considered reliable for assessing prenatal depression. The concurrent criterion validity was determined through cross tabulating the EPDS-L score against the actual prenatal depressive status based on the DSM-5 criteria as the gold standard.

The same hundred mothers were screened for depression using both the EPDS-L and DSM 5 criteria for MDD. Using the DSM 5 criteria, 35/100 mothers turned positive for depression, while 22/100 mothers screened positive for depression using the EPDS-L. The sensitivity of the EPDS-L was 62.86%; this implies that the screening tool is able to identify 62.86% of mothers with depression correctly. Specificity of the EPDS-L was 100%, thus the screening tool is able to identify 100% of mothers without depression correctly.

The Positive predictive value was 100%, implying that for those who tested positive for depression, 100% truly had the depression while the Negative predictive value 83.33%, implying that for those who tested negative, 83.33% truly did not have the depression (Tables 2 and 3).

**Figure 1:**
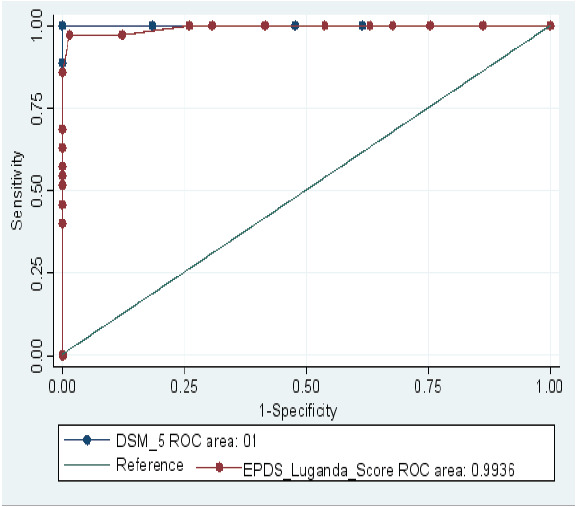
Receiver Operating Curve of the adapted Luganda version of the Edinburgh Postnatal Depression scale

The Area Under the Receiver Operating Characteristic curve (AUROC) was plotted to confirm whether the cut off level is optimum to define threshold level for depressive symptom using the EPDS-L. The sum total EPDS-L score was computed against the DSM-5 status and the levels of sensitivity and I-specificity was assessed for each EPDS-L score as seen in the figure 1 above. The Area Under the Receiver Operator Characteristic curve for the EPDS-L was 0.99 [95% CI: 0.98-1.00]. This suggests a 99% chance that the EPDS-L correctly distinguishes a mother with prenatal depression from one without it.

EPDS-L cut off points from nine (9) to 17 were analyzed for sensitivity and specificity in comparison with the DSM-5 criteria for MDD, as shown in Table 4. Notably, an EPDS-L cut off score of ≥10 has the closest sensitivity and specificity to the DSM-5 criteria for MDD (Table 4).

**Table 4:** Sensitivity and Specificity of different cut off levels of the adapted EPDS-L against DSM-5

CUT OFF		
	Sensitivity	Specificity
9	97.14%	87.69%
10	97.14%	98.46%
11	85.71%	100.00%
12	68.57%	100.00%
13	62.86%	100.00%
14	57.14%	100.00%
15	54.29%	100.00%
16	51.43%	100.00%
17	45.71%	100.00%

## Discussion

We conducted this study to adapt and validate the Luganda version of the Edinburgh Postnatal Depression scale for screening prenatal depression among pregnant women in the antenatal clinic at Kawempe National Referral Hospital in Uganda.

### Interpretation of findings

#### Adaptation of the EPDS-L

In this study, we followed the ISPOR taskforce guidelines during the adaptation of the EPDS-L 29. There are alternative methods for cross-cultural adaptation of Patient Reported Outcome tools such as the Delphi method 30, Modified Delpih Technique 31 and Nominal Group Technique 32 as applied in different settings. We however chose the ISPOR taskforce guidelines for logistical reasons. A review of the different methods involving experts found relative non-superiority, and that comparable results were obtained 33.We undertook adaptation of the EPDS-L with language experts from Makerere University.

#### Validation of the EPDS-L

The Cronbach's alpha coefficient of 0.8515 obtained is similar to findings of 0.86 in India 14. Validation of both the Indian version and the Luganda version was undertaken in LMIC settings in a similar sample of prenatal mothers. However, the Indian version was validated in comparison to the Patient Health Questionnaire-9 (PHQ-9) which is equally metric just like the EPDS.

We initially considered a cut of 13 while validating the EDPS-L. This was because in the validation of original English EPDS tool by Cox et al, a cut-off of 12/13 was found to identify all women with Major depressive disorder 12. However, this was done in a postpartum population of 84 women. Our study however does not differentiate between best cut off for minor and major depression. Murray at al attempted to differentiate the best cut off for either minor or major prenatal depression but concluded a cut-off of 12/13 was best for either 34. Additionally, a cut-off of 13 had been applied in a Ugandan prenatal depression prevalence study before 4. This particular study by Ndege et al applied the English EPDS. Further analysis of our data demonstrated that a cut-off of 10 had the best psychometric properties in screening prenatal depression. This is consistent with research done in Nigeria 35, which did validation among only late trimester women, and considered the MINI International Neuropsychiatric Interview as the gold standard. Research conducted in Europe 36 and Asia 37 found similar findings that considered a lower cut-off of 10. Perhaps different settings should undertake validation studies to ascertain their best-fit screening cut-off^38^.

#### Implications for practice, including the intended use and clinical role of the index test

The EPDS-L can be applied by any health care provider including primary health care workers to screen for prenatal depression. The EPDS-L should only be applied as a screening tool, whose result should prompt further inquiry if 10 or more. Further interventions include referral to psychiatric care or higher-level health care. The DSM-5 criteria for MDD remains the gold standard diagnostic and should be used to confirm the diagnosis.

#### Strengths

The study provides a much needed prenatal depression screening tool tailored for application by even the lowest-level health care provider in a LMIC. Given that the EPDS-L is written in Luganda, it maybe self-administered by patients who can read Luganda.

## Limitations

One of the limitation of this study is that the adapted EPDS-L was validated in a sample at national referral hospital. This sample may not be representative of the general population, or patients at lower level health care points. Utility of the EPDS-L is limited to only Luganda-speaking parts of Uganda. As such, utility in other regions may be limited by language barrier

## Conclusion and recommendation

The adapted EPDS-L can reliably screen prenatal depression at cut -off of 13 or more. The tool performs better at a cut- off of 10.

## Recommendations

Incorporation of the EPDS-L in all patient files of Luganda-speaking mothers should be considered. Validation of the EPDS-L at lower level health care to ensure both application by the undertaken to increase coverage.
